# An Integrated Imaging and Circulating Biomarker Approach for Secondary Tricuspid Regurgitation

**DOI:** 10.3390/jpm10040233

**Published:** 2020-11-16

**Authors:** Georg Spinka, Philipp E. Bartko, Gregor Heitzinger, Eliza Teo, Suriya Prausmüller, Henrike Arfsten, Noemi Pavo, Max-Paul Winter, Julia Mascherbauer, Christian Hengstenberg, Martin Hülsmann, Georg Goliasch

**Affiliations:** 1 Department of Internal Medicine II, Medical University of Vienna, 1090 Wien, Austria; georg.spinka@meduniwien.ac.at (G.S.); philippemanuel.bartko@meduniwien.ac.at (P.E.B.); n1542192@students.meduniwien.ac.at (G.H.); suriya.prausmueller@meduniwien.ac.at (S.P.); henrike.arfsten@meduniwien.ac.at (H.A.); noemi.pavo@meduniwien.ac.at (N.P.); max-paul.winter@meduniwien.ac.at (M.-P.W.); julia.mascherbauer@meduniwien.ac.at (J.M.); christian.hengstenberg@meduniwien.ac.at (C.H.); martin.huelsmann@meduniwien.ac.at (M.H.); 2 Department of Cardiology, Royal Melbourne Hospital, Melbourne 3144, Australia; eliteo@gmail.com

**Keywords:** HFrEF, secondary tricuspid regurgitation, cardiac biomarkers, endothelin, atrial natriuretic peptide, echocardiographic imaging prognosis

## Abstract

Secondary tricuspid regurgitation (sTR) is frequent among patients with heart failure with reduced ejection fraction (HFrEF), however it confers considerable diagnostic challenges. The assessment of neurohumoral activation may constitute a valuable supplement to the current imaging-based diagnostic process. This study sought to investigate the expression of complementary biomarkers in sTR and to evaluate the effectiveness of integrating their assessment into the diagnostic process. We enrolled 576 HFrEF patients recording echocardiographic and biochemical measurements, i.e., N-terminal pro-B-type natriuretic peptide, mid-regional pro-atrial natriuretic peptide (MR-proANP), mid-regional pro-adrenomedullin, C-terminal pro-endothelin-1 (CT-pro-ET1), and copeptin. Plasma levels of the aforementioned neurohormones were significantly elevated with increasing sTR severity (*p* < 0.001 for all). CT-pro-ET1 and MR-proANP were the closest related to severe sTR (adj. OR 1.46; 95%CI 1.11–1.91, *p* = 0.006 and adj. OR 1.45, 95%CI 1.13–1.87, *p* = 0.004, respectively). In patients with moderate-to-severe sTR, adding selected biomarkers (i.e., CT-pro-ET1 and MR-proANP) resulted in a substantial improvement in the discriminatory power regarding long-term mortality (C-statistic: 0.54 vs. 0.65, *p* < 0.001; continuous NRI 57%, *p* < 0.001). Circulating biomarkers closely relate to sTR severity and correlate with hemodynamic and morphologic mechanisms of sTR. Specifically, MR-proANP and CT-pro-ET1 are closely linked to the presence of severe sTR, and a combined assessment with the guideline recommended echocardiographic grading significantly improves individual risk stratification.

## 1. Introduction

Secondary tricuspid regurgitation (sTR) is frequently seen in patients with heart failure with reduced ejection fraction (HFrEF) and is independently associated with excess mortality despite guideline directed therapy (GDT) [1,2]. sTR is a complex disease, emerging due to structural alterations of the valvular, ventricular, and atrial geometry involving annular dilatation and leaflet tethering. Assessment of TR severity by echocardiographic imaging still incurs considerable challenges caused by the impaired visualization of the right heart, often leading to an underestimation of lesion severity. Moreover, despite current guideline recommendations demanding an integrated approach for the assessment of tricuspid regurgitation (TR) and the rising interest for percutaneous treatment strategies for severe sTR, this entity is still underdiagnosed [3,4,5,6,7,8]. Additional tools for improved identification and risk assessment of severe sTR remain an unmet clinical need to be able to account for the competing risks of an interventional procedure and the potential benefit of reducing long-term mortality.

Circulating biomarkers and their prognostic impact have been extensively studied in the context of chronic heart failure [9,10]. In HFrEF, volume overload of the dysfunctional ventricles and the vascular system induces wall stress, thereby promoting local and systemic neurohumoral activation, leading to further remodeling at a molecular and morphologic level. N-terminal pro-B-type natriuretic peptide (NT-proBNP) and mid-regional pro-atrial natriuretic peptide (MR-proANP) are released in response to an alteration in transmural pressure of the myocardium and have been associated with volume overload in heart failure and valvular regurgitation [11,12,13]. C-terminal pro-endothelin-1 (CT-pro-ET1) reflects the activity of Endothelin-1, which has previously been reported as a marker for venous congestion and increased pulmonary pressures [14,15,16]. Copeptin, a peptide derived from the precursor of vasopressin, has been associated with increased mortality in advanced heart failure patients [17,18]. Finally, mid-regional pro-adrenomedullin (MR-proADM) is a peptide reflecting the actions of adrenomedullin, a potent vasodilator that has been associated with heart failure and progressive mitral regurgitation [11,19,20]. These neurohumoral biomarkers therefore illustrate the hemodynamic state from a cardiac perspective as well as from a systemic vascular point of view.

Despite the rising awareness of these neurohumoral pathways in the setting of heart failure (HF), data on the relationship with sTR are scarce, and it remains unclear which established circulating biomarkers directly relate to sTR severity. As the diagnosis of severe sTR by echocardiographic imaging can be challenging, neurohormones indicative of the additional risk of this lesion might serve as a valuable supplement, especially in diagnostically difficult patients. Moreover, as minimally invasive therapies arise for the treatment of the tricuspid valve, the ability to precisely identify severe sTR for optimal and timely intervention is necessary and requires certain additional objective parameters. We therefore sought to investigate the association of a prespecified set of complementary neurohumoral biomarkers with the disease spectrum of sTR in HFrEF to assess their capability in identifying patients with severe sTR and to investigate their potential to improve individual risk stratification with regards to long-term mortality.

## 2. Materials and Methods

### 2.1. Study Population

We included consecutive patients with HFrEF at the heart failure outpatient clinic of the Vienna General Hospital, a university-affiliated tertiary center in this observational, non-interventional study. HFrEF was defined as a history of left ventricular (LV) ejection fraction below 40% paired with HF signs or symptoms in agreement with the latest HF guidelines [21]. Patients with primary TR or more-than-mild aortic or mitral stenosis or congenital heart disease were excluded. The patients were recruited in a clinically stable, non-decompensated condition to account for the dynamic nature of functional regurgitation. All-cause mortality was chosen as the primary endpoint and retrieved via inquiry of the Austrian Death Registry. The study protocol adheres to the Declaration of Helsinki and was approved by the Ethics Committee of the Medical University of Vienna with the ethical approval code 728/2007.

### 2.2. Clinical Measurements and Follow-Up

A clinical exam at baseline included the acquisition of medical history as well as current medication status, an electrocardiogram, and a transthoracic echocardiogram. Cardiovascular risk factors were documented in agreement with the respective guidelines [22]. To avoid a bias by treatment changes during follow-up, dosages of medical therapy were cautiously up-titrated to the maximal recommended/tolerated dose from the ESC guidelines before index echocardiography [21].

### 2.3. Laboratory Measurements and Hemodynamic Assessment

Venous blood samples were drawn from all patients at index time in order to analyze routine laboratory parameters according to the laboratory’s standard procedure. We carefully chose specific neurohormones that have either already proven their prognostic value or are promising to give new insights into the pathophysiology of HF and sTR, as previously described [23]. MR-proANP, MR-proADM, CT-proET-1, and Copeptin were measured in ethylenediaminetetraacetate (EDTA) plasma using specific sandwich immunoassays (BRAHMS, Hennigsdorf/Berlin, Germany). NT-proBNP measurements were performed in heparin plasma using the Elecsys Systems (Roche Diagnostics, Mannheim, Germany). We recorded invasive hemodynamic assessment in all patients with an indication to undergo right heart catheterization at index time. The investigation was performed using a 7F-Swan-Ganz Catheter (Edwards Lifesciences, Irvine, CA, USA) via femoral or jugular access. For recording of the pressures, we averaged eight measurements over eight consecutive cardiac cycles using CathCorLX (Siemens AG, Berlin, Germany).

### 2.4. Echocardiographic Assessment

All patients underwent a standard echocardiographic exam at index time recorded with commercially available equipment (Vivid5 and Vivid7, GE Healthcare, Little Chalfont, United Kingdom). Echocardiographic recordings were assessed by multiple experienced echocardiographers of the Echocardiography Laboratory of the Cardiology Department at the Medical University of Vienna. Examiners were entirely blinded to all hemodynamic and biochemical parameters as well as outcome assessment. Measurements were reported as an average of three beats for patients with sinus rhythm and ten beats for patients with atrial fibrillation or flutter in accordance with current recommendations [24]. Cardiac chambers were quantified using diameters in standard four- and two-chamber views and LV ejection fraction was calculated using the biplane Simpson method. Semi quantitative assessment of right ventricular function (RVF) was performed using multiple acoustic windows and graded as mild, mild-to-moderate, moderate, moderate-to-severe, and severe in accordance with the current recommendations [8]. TR was quantified and classified in an integrated approach comprising the morphology of the tricuspid valve as well as the visual assessment of both the width of the proximal regurgitant jet and the proximal flow convergence by color Doppler imaging. Present valvular regurgitation and stenosis were graded as mild, moderate, and severe according to the current guidelines [5]. Systolic pulmonary artery pressure (sPAP) was calculated by adding the estimated central venous pressure to the TR peak systolic gradient. 

### 2.5. Statistical Analysis

Discrete data were presented as count and percentage and compared by the χ^2^-test. Continuous data were presented as median and interquartile range (IQR) and analyzed using the Kruskal–Wallis test. Cox proportional hazard regression analysis was applied to determine the effect of TR severity on long-term mortality. To account for potential confounding effects, we formed a clinical confounder cluster encompassing age, gender, kidney function, and etiology of HF. Harrell C statistic was used to assess the discriminatory power of the respective variables. The improvement in individual risk prediction was analyzed by the net reclassification improvement (NRI). Univariable logistic regression analysis was used to assess the association between levels of neurohormones and the presence of severe sTR. Forward selection analysis was performed to determine the neurohormones with the strongest association with severe sTR. In a multivariable regression analysis, the selected parameters were adjusted for potential clinical confounders, i.e., age, kidney function, the presence of (≥moderate) mitral regurgitation, LV end-diastolic diameter, RV end-diastolic diameter (RVEDD), left ventricular function (LVF), and RVF for each biomarker. Results are displayed as odds ratios (OR) for a 1 standard deviation (SD) increase in continuous variables with the respective 95% confidence interval (CI). The discriminatory power of the different neurohormones was assessed by receiver operating characteristic curve (ROC) analysis. The correlation between neurohormones and invasively assessed hemodynamic as well as echocardiographic parameters was analyzed calculating Pearson-r correlation coefficient and displayed using a correlation plot. Colored fields indicate statistical significance, blue color indicates direct and red color indicates indirect correlation; the shade of the color indicates the correlation coefficient. Two-sided *p*-values < 0.05 were used to indicate statistical significance. The SPSS 25.0 (IBM Corp) and R-Statistics (R-Foundation) software packages were used for all statistical analyses.

## 3. Results

### 3.1. Baseline Characteristics

We included a total of 576 HFrEF patients with a median age of 58 years (IQR 50–64), 83% of patients were male. LVF was severely reduced in 56% of patients (*n* = 325) with a median LV ejection fraction of 27%. Forty-one percent of patients (*n* = 236) were in New York Heart Association (NYHA) functional class III, and 21% were in NYHA class IV (*n* = 121), representing a high-risk heart failure population. The etiology of HF was ischemic in 39% of patients (*n* = 225). Heart failure therapy was well established at index time, with 96% of patients (*n* = 551) receiving RAS-antagonists up-titrated to a median dosage of 100% of the maximal guideline recommended dose, 71% (*n* = 410) receiving beta-blockers up-titrated to a median dosage of 50% of the maximal guideline recommended dose, and 33% (*n* = 189) received mineral corticoid receptor antagonists. Hemodynamics were available in 150 patients. Detailed baseline characteristics of the entire study population are displayed in Table 1.

### 3.2. Baseline Characteristics According to Severity of Tricuspid Regurgitation

Detailed baseline characteristics of the entire study population according to TR severity are presented in Table 1. Moderate sTR was observed in 136 patients (24%) and severe sTR in 63 patients (11%). With increasing sTR severity, kidney function declined (*p* < 0.001) whereas levels of NT-proBNP (*p* < 0.001) and NYHA class (*p* < 0.001) significantly increased. Morphologically, sTR severity was closely related to increasing RVEDD [no/mild sTR: 34 mm (IQR 30–38); moderate sTR: 41 (IQR 35–45); severe sTR: 44 (40–49)], right atrial (RA) diameters [no/mild sTR: 55 mm (IQR 50–62); moderate sTR: 65 (IQR 58–71); severe sTR: 70 (IQR 60–77)], and systolic pulmonary artery pressure (sPAP) (*p* < 0.001). RVF declined with increasing sTR severity (*p* < 0.001).

### 3.3. Severity of Tricuspid Regurgitation and Outcome

Over a 5-year follow-up period, 251 patients (44%) died. sTR severity was associated with long-term mortality in the crude cox regression analysis with an HR of 1.57 (95% CI 1.33–1.84; *p* < 0.001). The results remained significant after adjustment for our clinical confounder model encompassing age, gender, kidney function, and etiology of HF with an adjusted HR of 1.59 (95% CI 1.35–1.88; *p* < 0.001).

### 3.4. Activation of Neurohormones in Severe Tricuspid Regurgitation

**Figure 1:**
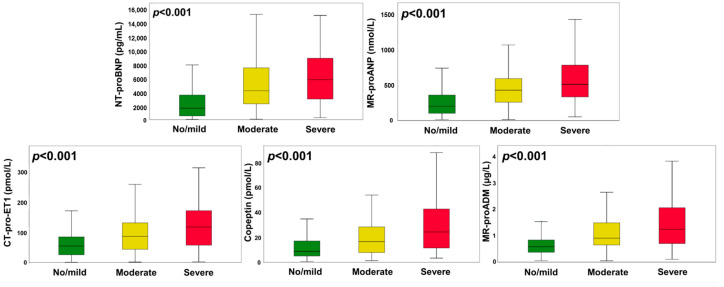
Neurohumoral profiles in HFrEF patients with no/mild, moderate, or severe secondary tricuspid regurgitation. Levels are displayed as Tukey boxplots. Comparisons between different degrees of tricuspid regurgitation were analyzed by the Kruskal–Wallis test. CT-pro-ET1, C-terminal pro-endothelin-1; MR-proADM, mid-regional pro-adrenomedullin; MR-proANP, mid-regional pro-atrial natriuretic peptide; NT-proBNP, N-terminal pro-B-type natriuretic peptide.

CT-pro-ET1, MR-proANP, NT-proBNP, MR-proADM, and Copeptin exhibited a strong association with TR and were significantly elevated with increasing lesion severity (*p* < 0.001 for all). Detailed neurohormonal profiles of all aforementioned variables according to the degree of TR are displayed in Figure 1.

We further observed a strong association between increased neurohumoral activation and the presence of severe sTR in the univariable logistic regression analysis. Increasing levels of CT-pro-ET1, MR-proANP, NT-proBNP, and Copeptin were associated with severe sTR with an OR for a 1-SD increase of 2.08 (95%CI 1.65–2.63, *p* < 0.001), 1.80 (95%CI 1.44–2.24, *p* < 0.001), 1.63 (95%CI 1.34–1.98, *p* < 0.001), and 1.60 (95%CI 1.31–1.95, *p* < 0.001), respectively. In contrast, MR-proADM was not related to severe sTR in our analysis. ROC analysis revealed an area under the curve of 0.75 for NT-proBNP, 0.70 for CT-pro-ET1, and 0.77 for MR-proANP. We then used a forward stepwise selection approach including all investigated neurohormones and identified that CT-pro-ET1 (*p* < 0.001) and MR-proANP (*p* = 0.008) were closest related to severe sTR. The associations between severe sTR and CT-pro-ET1 (OR 1.46, 95%CI 1.11–1.91, *p* = 0.006) and MR-proANP (OR 1.45, 95%CI 1.13–1.87, *p* = 0.004) remained virtually unchanged after multivariable adjustment for a clinical confounder model encompassing echocardiographic parameters and traditional clinical risk factors. Detailed results of the univariable and multivariable logistic regression analysis are displayed in Table 2. By means of individual outcome in patients with moderate to severe sTR, adding the selected biomarkers (i.e., CT-pro-ET1 and MR-proANP) resulted in a substantial improvement of the discriminatory power with regards to long-term mortality measured by Harrel C statistic (moderate to severe sTR: 0.54 vs. moderate to severe sTR + biomarkers: 0.65; *p* < 0.001). Correspondingly, an improvement in individual risk stratification with combined assessment of moderate to severe sTR and the selected biomarkers (i.e., CT-pro-ET1 and MR-proANP) was confirmed by a significant improvement of the NRI with 57% for mortality (*p* < 0.001).

### 3.5. Association of Neurohumoral Activation with Hemodynamic and Morphologic Characteristics

**Figure 2:**
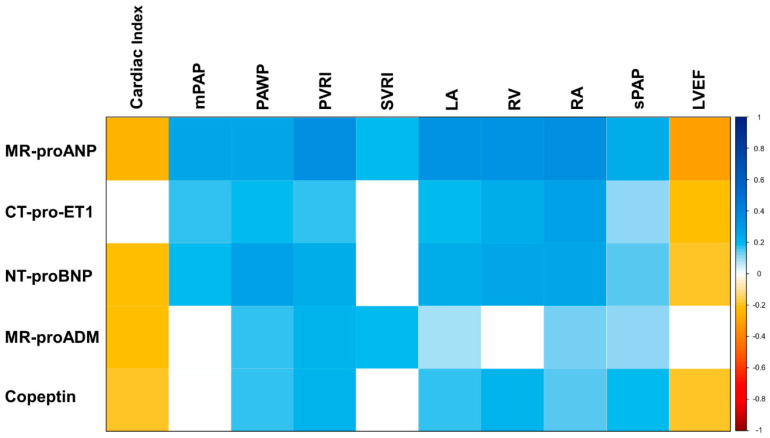
Correlogram investigating the correlation between neurohumoral activation and invasively measured hemodynamic parameters in HFrEF patients with secondary tricuspid regurgitation. Colored fields indicate statistical significance, blue color indicates direct correlation, red color indicates indirect correlation, the shade of the color indicates the correlation coefficient calculated by Pearson-r and referenced in the adjacent scale. CT-pro-ET1, C-terminal pro-endothelin-1; LA, left atrial diameter; LVEF, left ventricular ejection fraction; mPAP, mean pulmonary artery pressure; MR-proADM, mid-regional pro-adrenomedullin; MR-proANP, mid-regional pro-atrial natriuretic peptide; NT-proBNP, N-terminal pro-B-type natriuretic peptide; PAWP, pulmonary artery wedge pressure; PVRI, pulmonary vascular resistance index; RA, right atrial diameter; RV, right ventricular diameter; sPAP, systolic pulmonary artery pressure; SVRI, systemic vascular resistance index.

To further elucidate the mechanism of action relating increased neurohormones to the presence of severe sTR, we performed a correlation analysis with echocardiographic and invasively assessed hemodynamic parameters. The median left atrial (LA) diameter was 64 mm (IQR 57–71), median RA diameter was 58 mm (IQR 52–66), and median RVEDD was 36 mm (IQR 31–42). With regards to hemodynamic assessment, the median pulmonary artery wedge pressure (PAWP) was 23 mmHg (IQR 20–26), median mean pulmonary artery pressure (mPAP) was 38 mmHg (IQR 31–43), and mean pulmonary vascular resistance (PVRI) was 645 dyn∙s/cm^5^ (IQR 480–811). Our data suggest strong correlations between MR-proANP, CT-pro-ET1, and NT-proBNP with RA and LA diameter (*p* < 0.001 for all), RVEDD (*p* < 0.001 for all) as well as systolic pulmonary artery pressure. Moreover, we observed significant correlations between those neurohormones and important hemodynamic parameters of the right heart, namely PAWP and PVRI. Detailed results of the correlation analysis are displayed in Figure 2.

## 4. Discussion

This large-scale observational study describes for the first time an association between sTR severity and neurohumoral activation in HFrEF patients. The results specifically highlight MR-proANP and CT-pro-ET1 as strongly related to sTR severity and as powerful predictors for the presence of severe sTR. More importantly, individual risk stratification regarding mortality in sTR showed a significant improvement by adding these biomarkers to echocardiographic grading in patients with sTR. This has direct clinical implications as neurohumoral assessment can complement the assessment of secondary valve lesions that is currently exclusively imaging-based and thereby potentially impact therapeutic decision making.

### 4.1. Neurohumoral Activation in Secondary Tricuspid Regurgitation

The prognostic impact of activated neurohumoral pathways in chronic HF has been widely demonstrated [9,10]. HF is a syndrome with different cardiac and extracardiac systems involved, but it remains a conundrum whether, and if so, which circulating biomarker is closest related to distinct pathophysiologic mechanisms. Some neurohormones such as atrial natriuretic peptide, B-type natriuretic peptide, Endothelin-1, and Adrenomedullin are primarily associated with hemodynamic alterations of the heart and the vasculature system [19,25]. As sTR strongly depends on hemodynamic changes, it appears obvious that measurement of sTR and these circulating biomarkers are closely related. However, it remains unclear whether this information is redundant or complementary.

With progressive HF, patients develop pulmonary hypertension, which is thought to be the main driver of right ventricular dilatation and closely related to the development of sTR [26]. Regurgitation leads to atrial volume overload and dilatation. Intervention soon after this point is crucial, as progressive backward pressure leads to hepatic and renal failure [15,27], up to now without any available therapy. The progression in the continuum of backward failure can be well-documented quantitatively by imaging methods [28]. Measurements of the RA, right ventricle (RV), TR, and afterload can be performed, but the value in providing integrated information about severity and prognosis is limited, as these measurements are interrelated as well as dependent on other variables (e.g., volume load, blood pressure, or heart rate).

### 4.2. Neurohormones as Indicators of Morphologic and Hemodynamic Changes in sTR

Physiologically, the active hormones atrial natriuretic peptide (ANP) and B-type natriuretic peptide (BNP) are primarily released by cardiac myocytes in response to an alteration of transmural pressure following atrial and ventricular volume overload. Accordingly, our data show a strong correlation between the level of natriuretic peptides and echocardiographic parameters for cardiac chamber dimensions. MR-proANP has already been shown to be related to the size of the left atrium and ventricle in the context of secondary mitral regurgitation (sMR) [11,12], reflecting morphologic maladaptation as a result of valvular regurgitation on the atrial and ventricular myocardium [29]. In addition, in the presence of sTR as in our study, MR-proANP displayed the strongest correlation with atrial diameters of both sides, while NT-proBNP correlated best with diameters of the right and left atrium and ventricle. This is in accordance with the fact that ANP is primarily produced in the atria, while BNP is essentially built in the ventricles. Concerning hemodynamic assessment, MR-proANP shows a strong correlation with rising pulmonary and systemic resistance parameters. Considering RA size as surrogate and elevated pulmonary pressures as a possible cause of sTR, the correlation with MR-proANP possibly reflects the burden of sTR on the atrial myocardium.

CT-pro-ET1 is a stable parameter reflecting the activity of Endothelin-1, which is a potent peripheral vasoconstrictor produced by endothelial cells [16]. Different to this physiologic process, but previously described in patients with HF [30], our data imply that levels of CT-pro-ET1 are independent of SVRI, thus constituting a perfect marker for backward failure. Moreover, CT-pro-ET1 levels show a pronounced correlation with RA diameter. According to the literature, CT-pro-ET1 correlated well with increased pulmonary pressures and resistance, thus seems to specifically reflect the effects of increased afterload for the RV. This would be in accordance with reports defining it as a biomarker for venous congestion [14], often present in HFrEF and sTR and closely related to increased pulmonary pressures [15]. Considering elevated pulmonary pressures and enlarged RA diameters as indicators for the load of sTR, the strong correlation with CT-pro-ET1 possibly reveals this neurohormone as a biomarker for morphologic and hemodynamic maladaptation closest to sTR.

Our data show that elevated levels of all measured hormones such as CT-pro-ET1, MR-proANP, NT-proBNP and Copeptin were significant predictors for the presence of severe sTR. As the group of patients with severe sTR frequently presented with concomitant sMR and severely depressed LVF, it is arguable that the neurohumoral activation reflects the severity of the underlying advanced heart failure, which is supported by the inverse correlation with LV ejection fraction. Notwithstanding, the traditional and established heart failure biomarker NT-proBNP shows a strong relationship to sTR in the ROC analysis and has previously been associated with valvular regurgitation [23]. However, the aim of the present analysis was to display the set of variables that reflects sTR severity best. After stepwise forward selection and adjustment for a comprehensive confounder cluster including LVF and the presence of ≥moderate MR, CT-pro-ET1 and MR-proANP remained strong predictors for severe sTR, whereas NT-proBNP did not. It is possible that the well-established NT-proBNP reflects the burden of heart failure including valvular regurgitation in general, whereas CT-pro-ET1 and MR-proANP seem to specifically indicate the presence of sTR and therefore represent valuable diagnostic additions. Despite the obviously near pathophysiologic relationship between these biomarkers, left ventricular function, and valvular regurgitation, the present data imply that CT-pro-ET1 and MR-proANP are distinct predictors for the presence of severe sTR. Moreover, incorporating these markers with echocardiographic grading of sTR severity leads to significantly better calibration towards mortality than with imaging solely. As a more widely available parameter in clinical routine, NT-proBNP still appears as a reliable measure for the diagnosis of sTR if CT-pro-ET1 and MR-proANP are not available.

Interestingly, while MR-proADM infers prognostic impact for progressive mitral regurgitation [11], it was not able to predict severe sTR. The correlation with cardiac index and systemic vascular resistance was more pronounced than with pulmonary resistance parameters in our study. As a marker for forward failure [11], it seems plausible that MR-proADM is relevant in the context of sMR as a reflection of LV afterload, whereas it is not prognostic in sTR. Vice versa, CT-pro-ET1 reflects RV afterload and therefore represents the driver of sTR, whereas MR-proADM as a marker of LV afterload becomes neglectable. As natriuretic peptides are considered as more general indicators of atrial burden in secondary valvular regurgitation, it is possible that MR-proADM is indicative for sMR, while CT-pro-ET1 is a more specific biomarker for the presence of severe sTR.

### 4.3. Clinical Implications of Neurohumoral Activation in the Diagnosis of Severe sTR

On the basis of our results, the assessment of MR-proANP and CT-pro-ET1 could prove to be a valuable supplement in the management and risk estimation of patients with sTR. Current guideline recommendations demand an integrated approach to grade sTR severity, however these are based on imaging techniques solely [5]. The echocardiographic visualization of the right heart and its pathologies still inheres significant difficulties and often leads to an underestimation of tricuspid lesions. Moreover, the symptom spectrum of patients with sTR ranges from absent, i.e., in incidental findings during routine echocardiography, to severely pronounced, where patients present with peripheral edema, hepatomegaly, and ascites. Echocardiographic grading is not always consistent with the clinical condition, leading to uncertainty in the diagnosis and subsequent treatment guidance. Indeed, treatment options for these patients have been very limited in the past, with isolated tricuspid valve surgery representing the surgical intervention with the highest mortality risk in this field [31]. However, the precise diagnosis and the resulting treatment decisions are regaining importance in light of newly emerging transcatheter therapies for tricuspid valve interventions [32]. The potential to reliably differentiate between higher grades of sTR is essential to adequately select patients for timely intervention. Importantly in these patients, the additional information provided by the assessment of an objective, non-imaging-based parameter that reflects hemodynamic and morphologic maladaptation could be of value in decision making. In clinical practice, the assessment of these circulating biomarkers appears as an efficient and objective supplement to strengthen the suspicion for severe sTR, especially in patients with impaired imaging quality or diverging quantitative measures. Moreover, implementing CT-pro-ET1 and MR-proANP into a prediction model with current guideline recommended echocardiographic grading in patients with moderate to severe sTR results in considerable improvement in the individual risk stratification regarding patient prognosis. Thus, the combined assessment may be able to improve the management of patients with clinically meaningful sTR and to more precisely identify those with the highest risk of mortality, as they would benefit most from additional treatment options.

### 4.4. Limitations

The present study illustrates the experience of a single tertiary care center. Nevertheless, this assures a homogenous patient population undergoing imaging exams with consistent acquisition quality, while pursuing a coherent clinical routine. The patients were recruited and treated in the out-patient clinic of this tertiary center, therefore a decompensated state of HF at index echocardiography could be ruled out.

## 5. Conclusions

This long-term observational study is the first to assess neurohumoral activation in patients with sTR. Our data show distinctly elevated levels of CT-pro-ET1, MR-proANP, NT-proBNP, Copeptin, and MR-proADM across the disease spectrum of sTR and continuously rising levels with increasing TR severity. Specifically, CT-pro-ET1 and MR-proANP correlate with morphologic and hemodynamic parameters and are strong predictors for the presence of severe sTR. Importantly, the combined assessment of these circulating biomarkers and the current recommended echocardiographic grading offers complementary information and significant improvement in individual risk stratification towards long-term mortality in patients with sTR. Thus, they might complement the currently exclusively imaging-based assessment of secondary valve lesions and thereby potentially impact therapeutic decision making.

## Figures and Tables

**Table 1:** Baseline characteristics of the entire study population (*n* = 576) according to severity of secondary tricuspid regurgitation.

Baseline Characteristics	Total Study Population (*n* = 576)	No/Mild sTR (*n* = 377)	Moderate sTR (*n* = 136)	Severe sTR (*n* = 63)	*p*-Value
Age, median years (IQR)	58 (50–64)	57 (50–63)	58 (50–65)	60 (50–65)	0.391
Male sex, *n* (%)	476 (83)	317 (84)	110 (81)	49 (78)	0.391
BMI, kg/m^2^ (IQR)	26 (24–29)	27 (24–29)	26 (24–28)	26 (22–28)	**0.001**
Systolic blood pressure, mmHg (IQR)	115 (100–130)	120 (105–135)	109 (95–120)	100 (90–115)	**<0.001**
Ischemic etiology of HF, *n* (%)	225 (39)	160 (42)	49 (36)	16 (25)	**0.026**
Hypertension, *n* (%)	284 (49)	210 (56)	57 (42)	17 (27)	**<0.001**
Diabetes, *n* (%)	130 (23)	92 (24)	27 (20)	11 (17)	0.326
Hypercholesterolemia, *n* (%)	234 (41)	175 (46)	46 (34)	13 (21)	**<0.001**
Left bundle branch block, *n* (%)	112 (19)	80 (21)	23 (17)	9 (14)	0.872
Atrial fibrillation, *n* (%)	119 (21)	58 (15)	45 (33)	16 (25)	**<0.001**
NYHA functional class					**<0.001**
NYHA II, *n* (%)	153 (27)	121 (32)	24 (18)	8 (13)	
NYHA III, *n* (%)	236 (41)	151 (40)	62 (46)	23 (37)	
NYHA IV, *n* (%)	121 (21)	49 (13)	42 (31)	30 (48)	
Creatinine, mg/dL (IQR)	1.2 (1.0–1.4)	1.2 (1.0–1.3)	1.2 (1.0–1.5)	1.3 (1.1–1.7)	**<0.001**
Blood urea nitrogen, mg/dL (IQR)	20 (17–30)	20 (15–26)	23 (18–37)	30 (20–38)	**<0.001**
**Echocardiographic characteristics**					
Left ventricular end-diastolic diameter, mm (IQR)	64 (58–71)	63 (56–70)	64 (59–70)	66 (61–73)	**0.048**
Left ventricular function					
Moderately reduced (EF 30–40%), *n* (%)	159 (28)	120 (32)	32 (24)	7 (11)	**0.001**
Severely reduced (EF < 30%), *n* (%)	325 (56)	177 (47)	93 (68)	55 (87)	**<0.001**
Left ventricular ejection fraction, % (IQR)	27 (20–35)	30 (22–37)	26 (20–35)	22 (14–26)	**<0.001**
Left atrial diameter, mm (IQR)	64 (57–71)	61 (55–68)	69 (64–74)	72 (66–77)	**<0.001**
Right ventricular end-diastolic diameter, mm (IQR)	36 (31–42)	34 (30–38)	41 (35–45)	44 (40–49)	**<0.001**
Right ventricular function					
Moderately reduced, *n* (%)	23 (4)	4 (1)	6 (4)	13 (21)	**<0.001**
Severely reduced, *n* (%)	13 (2)	4 (1)	6 (4)	3 (5)	**0.030**
Right atrial diameter, mm (IQR)	58 (52–66)	55 (50–62)	65 (58–71)	70 (60–77)	**<0.001**
Mitral regurgitation (≥moderate), *n* (%)	193 (34)	71 (19)	73 (54)	49 (78)	**<0.001**
Systolic pulmonary artery pressure, mmHg (IQR)	46 (39–56)	41 (35–50)	50 (43–59)	54 (46–60)	**<0.001**
**Medication**					
RAS-antagonist, *n* (%)	551 (96)	363 (96)	128 (94)	60 (95)	0.559
Percent of maximal recommended dose, median %	100	100	100	100	0.15
Beta blockers, *n* (%)	410 (71)	274 (73)	96 (71)	40 (63)	0.324
Percent of maximal recommended dose, median %	50	50	50	44	0.66
Mineral corticoid receptor antagonist, *n* (%)	189 (33)	100 (27)	62 (46)	27 (42)	**<0.001**
Furosemide, *n* (%)	429 (75)	253 (67)	118 (87)	58 (92)	**<0.001**
**Device therapy**					
Implanted cardioverter defibrillator, *n* (%)	69 (12)	40 (11)	19 (14)	10 (16)	0.352
Pacemaker, *n* (%)	100 (17)	50 (13)	33 (24)	17 (27)	**0.002**
Cardiac resynchronization therapy, *n* (%)	55 (10)	41 (11)	11 (8)	13 (21)	0.250
**Hemodynamic characteristics**	N = 150	N = 150			
mPAP, mmHg (IQR)	38 (31–43)	39 (33–46)	36 (31–40)	38 (33–42)	0.194
PAWP, mmHg (IQR)	23 (20–26)	24 (21–26)	22 (20–26)	23 (21–28)	0.543
Cardiac Index, l/min/m^2^ (IQR)	1.8 (1.5–2.1)	1.8 (1.5–2.1)	1.9 (1.6–2.1)	1.7 (1.5–2.0)	0.512
Pulmonary vascular resistance, dyn∙s/cm^5^ (IQR)	635 (480–811)	699 (499–898)	595 (395–722)	600 (467–770)	0.142
Systemic vascular resistance, dyn∙s/cm^5^ (IQR)	2766 (2360–3368)	2905 (2480–3721)	2641 (2282–3057)	2746 (2452–3095)	0.131
**Neurohormones**					
NT-proBNP, pg/mL (IQR)	2360 (867–5163)	1632 (541–3510)	4131 (2262–7408)	5700 (2875–9083)	**<0.001**
MR-proANP, pmol/L (IQR)	275 (131–479)	202 (102–359)	429 (258–592)	510 (329–799)	**<0.001**
MR-proADM, nmol/L (IQR)	0.67 (0.42–1.06)	0.59 (0.37–0.84)	0.91 (0.65–1.50)	1.25 (0.62–2.12)	**<0.001**
Copeptin, pmol/L (IQR)	11.3 (5.8–21.8)	8.8 (5.0–17.2)	16.8 (7.9–28.7)	24.6 (11.4–43.9)	**<0.001**
CT-pro-ET1, pmol/L (IQR)	62 (31–106)	55 (26–85)	87 (44–132)	118 (56–173)	**<0.001**

Bold values indicate statistical significance. BMI indicates body mass index; CT-pro-ET-1 indicates C-terminal pro-endothelin-1; EF, ejection fraction; HF, heart failure; IQR, interquartile range; mPAP, mean pulmonary artery pressure; MR-proADM, mid-regional pro-adrenomedullin; MR-proANP, mid-regional pro-atrial natriuretic peptide; NT-proBNP, N-terminal pro-B-type natriuretic peptide; NYHA, New York Heart Association; PAWP, pulmonary artery wedge pressure; RAS, renin–angiotensin–aldosterone system.

**Table 2:** Univariable logistic regression analysis assessing risk factors for the presence of severe sTR. Forward selection analysis among neurohormones was performed to determine the neurohormones with the strongest association with severe sTR (i.e., CT-pro-ET1 and MR-proANP).

	Univariable Model					Selected Neurohormones		
	SD	OR	95% CI	*p*-Value	ROC	Adj. HR ^1^	95% CI	*p*-Value
Neurohormones								
CT-pro-ET1	65.3	2.08	1.65–2.63	**<0.001**	0.70	1.46	1.11–1.91	**0.006**
MR-proANP	347	1.80	1.44–2.24	**<0.001**	0.77	1.45	1.13–1.87	**0.004**
NT-proBNP	5452	1.63	1.34–1.98	**<0.001**	0.75			
Copeptin	23.7	1.60	1.31–1.95	**<0.001**	0.73			
MR-proADM	2.19	1.17	0.96–1.43	0.121	0.72			

Odds ratios (OR) refer to a 1 SD (standard deviation) increase in continuous variables. Bold values indicate statistical significance. CI indicates confidence interval; CT-pro-ET-1, C-terminal pro-endothelin-1; MR-proADM, mid-regional pro-adrenomedullin; MR-proANP, mid-regional pro-atrial natriuretic peptide; NT-proBNP, N-terminal pro-B-type natriuretic peptide; ROC, receiver operating characteristic curve. **^1^** Adjusted for age, kidney function, mitral regurgitation, left ventricular end-diastolic diameter, left ventricular function, right ventricular end-diastolic diameter, right ventricular function.
